# Thyroid hormone-induced cell-cell interactions are required for the development of adult intestinal stem cells

**DOI:** 10.1186/2045-3701-3-18

**Published:** 2013-04-01

**Authors:** Takashi Hasebe, Liezhen Fu, Thomas C Miller, Yu Zhang, Yun-Bo Shi, Atsuko Ishizuya-Oka

**Affiliations:** 1 Department of Biology, Nippon Medical School, 2-297-2 Nakahara-ku, Kosugi-cho, Kawasaki, Kanagawa, 211-0063, Japan; 2 Section on Molecular Morphogenesis, Program in Cellular Regulation and Metabolism (PCRM), Eunice Kennedy Shriver National Institute of Child Health and Human Development (NICHD), National Institutes of Health (NIH), Bethesda, Maryland, 20892, USA

**Keywords:** Adult organ-specific stem cell, Thyroid hormone receptor, Dedifferentiation, Apoptosis, Metamorphosis, *Xenopus laevis* and *tropicalis*, Cell-cell interaction, Matrix metalloproteinase, Extracellular matrix (ECM)

## Abstract

The mammalian intestine has long been used as a model to study organ-specific adult stem cells, which are essential for organ repair and tissue regeneration throughout adult life. The establishment of the intestinal epithelial cell self-renewing system takes place during perinatal development when the villus-crypt axis is established with the adult stem cells localized in the crypt. This developmental period is characterized by high levels of plasma thyroid hormone (T3) and T3 deficiency is known to impair intestinal development. Determining how T3 regulates adult stem cell development in the mammalian intestine can be difficult due to maternal influences. Intestinal remodeling during amphibian metamorphosis resembles perinatal intestinal maturation in mammals and its dependence on T3 is well established. A major advantage of the amphibian model is that it can easily be controlled by altering the availability of T3. The ability to manipulate and examine this relatively rapid and localized formation of adult stem cells has greatly assisted in the elucidation of molecular mechanisms regulating their formation and further revealed evidence that supports conservation in the underlying mechanisms of adult stem cell development in vertebrates. Furthermore, genetic studies in *Xenopus laevis* indicate that T3 actions in both the epithelium and the rest of the intestine, most likely the underlying connective tissue, are required for the formation of adult stem cells. Molecular analyses suggest that cell-cell interactions involving hedgehog and BMP pathways are critical for the establishment of the stem cell niche that is essential for the formation of the adult intestinal stem cells.

## Introduction

The intestinal epithelium is responsible for the principle physiological function of this organ: digestion and absorption of nutrients. Throughout adult life, the vertebrate intestinal epithelium undergoes self-renewal through the proliferation of the adult stem cells. In mammals, the stem cells are localized in the crypt of intestine while the absorptive epithelial cells, the most abundant epithelial cell type, and secretory cells are present along the villus of the crypt-villus axis [1-4]. As the stem cells proliferate in the crypt, their daughter cells migrate up along the crypt-villus axis and gradually differentiate into different types of epithelial cells, leading to the replacement of the entire epithelium once every 1–6 days in mammals [1,2,4].

While intestinal development occurs quite early during mammalian embryogenesis, the maturation of this adult epithelial self-renewing system takes place during the so-called postembryonic development [5-9], the perinatal period when plasma thyroid hormone (T3) level peaks [10,11]. While the underlying mechanism remains unclear, recent studies suggest that in mouse, the maturation involves the formation of adult stem cells that are distinct from the embryonic/neonatal intestinal stem cells [5,6,9,12]. In addition, T3 is important for the development and/or function of the adult stem cells. T3 deficiency or knockout of T3 receptors (TRs), which mediate the transcriptional effects of T3 on target genes, decreases the number of epithelial cells along the crypt-villus axis and proliferating crypt cells, leading to abnormal intestinal morphology [13-16]. Genetic studies in mouse suggest that TRα1, one of the two nuclear receptors for T3, controls intestinal development during maturation and also intestinal homeostasis in the adult by regulating the proliferation of intestinal stem cells [17-19].

Amphibian metamorphosis resembles postembryonic development in mammals [11,20]. This process is totally dependent on the presence of T3 [21-24]. Importantly, it can be easily manipulated by controlling the availability of T3 to the tadpoles via either inhibiting endogenous T3 synthesis or adding exogenous, physiological levels of T3 to the tadpole rearing water. This makes amphibian metamorphosis a superior model to study the developmental mechanisms *in vivo* when compared to mammalian postembryonic development, where maternal influences complicate the studies on the embryos/neonates.

**Figure 1:**
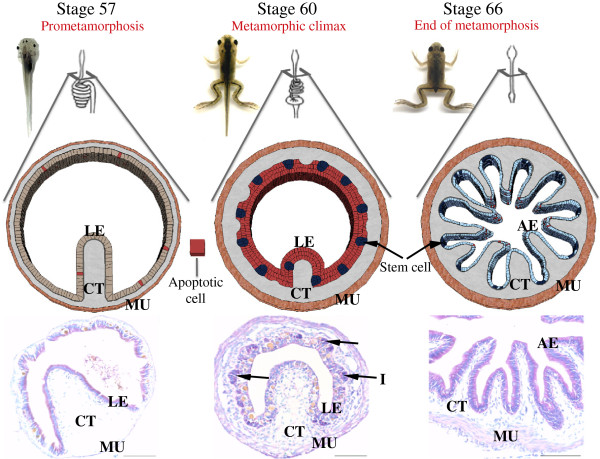
**Intestinal remodeling during *Xenopus *metamorphosis as a model to study the formation of adult stem cells during vertebrate development.** The illustration (top) depicts the straightening and shortening of the larval intestine during frog metamorphosis. The connective tissue (CT) and muscle (MU) layers increase in size during remodeling while the larval epithelial cells (LE) undergo apoptosis (red cells) or dedifferentiate into adult progenitor/stem cells (dark blue) which rapidly proliferate to form a more folded, mammalian-like epithelial surface with stem cells located at the troughs between epithelial folds. Cross-sections (below) of the intestine at different stages of *Xenopus laevis* development were stained with pyronin-Y (red staining) and methyl green (blue staining). During the early stage of metamorphosis (prometamorphosis), e.g., stage 57, the intestine is made of mostly a monolayer of larval epithelial cells with little connective tissue (CT) or muscles (MU) except within the single fold, the typhlosole, where CT is abundant. The epithelial cells are moderately stained red. At the climax of metamorphosis (stage 60), most of the larval epithelial cells undergo apoptosis and become stained much weaker. At this stage, strong pyronin-Y signals are strictly localized in the proliferating adult epithelial progenitor/stem cells within the islets (I, arrows). The CT and MU develop extensively during metamorphic climax. By the end of metamorphosis (stage 66), the adult epithelium with multiple folds is formed, with the adult stem cells localized to the trough of the epithelial fold, equivalent to the crypt in the adult mammalian intestine. LE: larval/tadpole epithelium, AE: adult epithelium, CT: connective tissue, MU: muscles, I: islets (clusters of proliferating adult epithelial progenitor/stem cells).

The remodeling of the intestine during amphibian metamorphosis resembles mammalian intestinal maturation. Like in mammals, the adult intestinal epithelium is constantly self-renewed, once every 2 weeks in *Xenopus laevis*[25]. The adult stem cells are localized at the bottom (trough) of the multiple folds (Figure 1), equivalent to the crypt in mammals, while the fully differentiated epithelial cells undergo apoptosis at the tip (crest) of the epithelial fold [25,26], again like the cell death at the tip of the villus in mammals. The formation of the adult intestine takes place during amphibian metamorphosis when circulating T3 level peaks, just like the maturation of the mammalian intestine during the perinatal period. In amphibians such as *Xenopus laevis* and *tropicalis*, the premetamorphic tadpole intestine is a simple tubular structure made of mostly a single layer of larval epithelial cells with little connective tissue or muscles except in the single fold, the typhlosole, where the connective tissue is abundant (Figure 1) [25,27,28]. During metamorphosis, the larval epithelial cells undergo T3-induced apoptosis. Concurrently, some larval epithelial cells escape cell death and instead undergo dedifferentiation to become the adult progenitor/stem cells (Figure 1), which eventually form an adult epithelium comprised of multiple folds surrounded by much thicker layers of connective tissue and muscles, resembling the adult mammalian intestine. Thus, intestinal metamorphosis offers a unique opportunity to investigate the mechanisms governing the formation of the adult intestinal stem cells during vertebrate development.

### A requirement for tissue-tissue interactions during intestinal metamorphosis

During metamorphosis the intestinal epithelium undergoes a dramatic change involving essentially the complete degeneration of the larval epithelium through apoptosis followed by *de novo* development of the adult epithelium (Figure 1) [27]. The other major tissues, the connective tissue and muscles, also change extensively, most noticeably the increase in the thickness of the tissue layers (Figure 1) [25,27,28]. Interestingly, a number of studies indicate that the changes in different tissues depend on tissue-tissue interactions, especially at the epithelium-connective tissue interface. First, the extracellular matrix (ECM) is known to influence cell fate and behavior through direct interactions with cells through cell surface receptors such as integrins and also by regulating the availability of extracellular signaling molecules such as growth factors [29-33]. The intestinal epithelium is separated from the underlying connective tissue by a special ECM, the basement membrane or basal lamina. In premetamorphic tadpoles or frogs, the basal lamina is thin but continuous. During metamorphosis, it becomes much thicker and amorphous [27,34,35]. This ECM appears to be more permeable as reflected by 1) the migration of macrophages from the connective tissue across the basal lamina to the epithelium, where they participate in the removal of the apoptotic cells [36], and 2) frequently observed contacts between proliferating adult epithelial progenitor/stem cells and fibroblasts in the connective tissue [35]. Thus, ECM remodeling likely plays an important role in intestinal remodeling by regulating cell-cell and cell-ECM interactions.

Second, studies using primary cultures of tadpole intestinal cells have provided direct support for a role of ECM in adult epithelial development. When isolated premetamorphic tadpole intestinal epithelial and fibroblastic cells were cultured *in vitro* on plastic dishes, T3 treatment led to proliferation of both cell types and at the same time caused the epithelial cells, but not the fibroblasts, to undergo apoptosis [37,38], resembling what occurs during metamorphosis. When the plastic dishes were coated with ECM proteins such as laminin and fibronectin, the T3-induced epithelial cell death was reduced [37]. These results suggest that ECM affects cell fate during metamorphosis. Since the basal lamina, the ECM that separates the epithelium and the connective tissue, is made of proteins secreted by both the epithelium and connective tissue, these findings suggest that ECM remodeling and changes in the connective tissue during intestinal metamorphosis can influence epithelial cell response to T3.

The extensive contacts between developing adult epithelial progenitor/stem cells and the fibroblasts in the underlying connective tissue at the climax of intestinal metamorphosis support the importance of cell-cell interactions for this process. *In vitro* organ culture studies have provided direct evidence to support an interdependence of epithelium and connective tissue for their respective changes during metamorphosis [39,40]. Of particular relevance to adult stem cell development is the observation that when anterior intestinal fragments of premetamorphic *Xenopus laevis* tadpoles were cultured *in vitro* in the presence of T3, the intestine underwent normal metamorphic changes, including larval epithelial apoptosis and the development of the adult progenitor/stem cells [39]. In contrast, when posterior intestinal fragments were cultured similarly *in vitro*, only larval cell death but no adult epithelial progenitor/stem cell formation was observed. The major difference between the anterior and posterior small intestine in *Xenopus laevis* tadpoles is the presence of the typhlosole, where the connective tissue is abundant, in the anterior but not posterior intestine. The formation and maturation of adult epithelial tissue occurs initially at the anterior end of the intestine during metamorphosis [41]. These suggest that the connective tissue is important for the development of the adult epithelium. Consistently, when posterior intestinal epithelium was recombined with the rest of the intestine (the non-epithelium) of the anterior intestine to generate a recombinant organ culture, T3 treatment now could produce adult progenitor/stem cells. Conversely, when anterior epithelium was recombined with posterior non-epithelium, only epithelial cell death was induced by T3 [39]. Thus, the non-epithelial cell layers in the intestine play an essential role, likely by contributing to the formation of a niche with appropriate signals for the induction of epithelial stem cells during metamorphosis.

### Gene regulation by T3 during intestinal metamorphosis

T3 functions by regulating gene transcription through TRs, which are DNA binding transcription factors belonging to the nuclear receptor superfamily [42-45]. Notably, studies on *Xenopus laevis* metamorphosis have shown that TR mediates target gene regulation by T3 during development and is both necessary and sufficient for amphibian metamorphosis [23,24,46-55]. Mechanistically, TR functions by recruiting cofactor complexes to T3 target genes to regulate transcription and many TR-interacting proteins have been characterized biochemically and in cell cultures [43,56-76]. These cofactor complexes function in part through histone modification and chromatin remodeling [73-77]. Molecular and genetic studies on *Xenopus* development have shown that in premetamorphic tadpoles, unliganded TR recruits corepressor complexes to endogenous T3-inducible genes to repress their expression and prevent premature metamorphosis [48,78-80]. When T3 is available, the binding of T3 to TR leads to the release of the corepressor complexes and the recruitment of the coactivator complexes. This results in gene activation and metamorphic transformation of different organs/tissues [81-86].

Many of the genes regulated by T3 during *Xenopus laevis* intestinal metamorphosis have been identified by various methods over the years [87-91]. Some are directly regulated at the transcription level by TR while others are indirectly regulated, downstream T3-response genes involved in intestinal transformation. Importantly, gene ontology analysis of genome-wide microarray data revealed that T3 response genes are highly enriched within functional categories that correlate well with both larval cell death and adult stem cell development in the intestine [88,90], supporting the involvement of these genes during intestinal metamorphosis. Interestingly, when expression of some of these genes were analyzed during intestinal development in *Xenopus tropicalis*, a species highly related to *Xenopus laevis*, the regulation patterns were found to be conserved [92-97], consistent with the similar changes in the intestine during *Xenopus tropicalis* metamorphosis [98]. Furthermore, for most of the genes that are highly upregulated in the *Xenopus laevis* intestine only at the climax of metamorphosis (stage 61), their mouse homologs also have their peak levels of expression in the intestine within the first 2 weeks after birth [90,99], when the mouse intestine matures into the adult form and plasma T3 levels are high. Thus, there is likely a conservation of T3-dependent gene regulation programs in the formation of the adult intestine in vertebrates.

### T3 regulation of cell-cell and cell-ECM interactions during intestinal metamorphosis

#### Tissue-specific requirements for T3 action in adult stem cell development

Organ culture studies as reviewed above have suggested that the non-epithelium, most likely the connective tissue, is required for T3-induced formation of adult progenitor/stem cells during intestinal metamorphosis. Gene expression analyses have shown that many genes are regulated by T3 in the epithelium or connective tissue or both. To investigate whether T3 actions in the epithelium and non-epithelium have specific roles in adult intestinal stem cell development, we made use of a transgenic *Xenopus laevis* line that expresses a dominant positive TR under the control of a heat shock-inducible promoter for organ culture studies. The dominant positive TR could not bind to T3 but functioned as a constitutively liganded TR to induce metamorphosis when expressed after heat shock treatment of premetamorphic tadpoles [49]. Thus, to selectively activate T3 signaling in the epithelium or the non-epithelium (the rest of the intestine), we could recombine the epithelium or the non-epithelium of the intestine of premetamorphic transgenic tadpole with non-epithelium or the epithelium of wild type siblings, respectively, and subject the recombinants to heat shock treatment. This led to the expression of the dominant positive TR in the transgenic tissues while the endogenous wild type TR remained unliganded in both the wild type and transgenic tissues. Using such an approach, we recently showed that expression of the dominant positive TR in the epithelium alone led to the formation of epithelial cells expressing Sonic hedgehog (Shh), which is highly expressed in the developing adult progenitor/stem cells during intestinal metamorphosis, as well as larval epithelial apoptosis, mimicking natural development [100]. Expression of the dominant positive TR in the non-epithelium, however, did not lead to the formation of epithelial cells expressing Shh, although larval epithelial apoptosis was induced. Interestingly, expression analyses of markers for adult mammalian intestinal stem cells showed that the Shh positive cells formed upon dominant positive TR expression in the epithelium alone were not true stem cells, and expectedly, such recombinant cultures failed to form differentiated adult epithelium after extended culturing. On the other hand, when dominant positive TR was expressed in both the epithelium and non-epithelium, the Shh positive cells also expressed the stem cell markers of adult intestine and the corresponding recombinant organ cultures developed differentiated adult epithelium after extended culturing [100].

**Figure 2:**
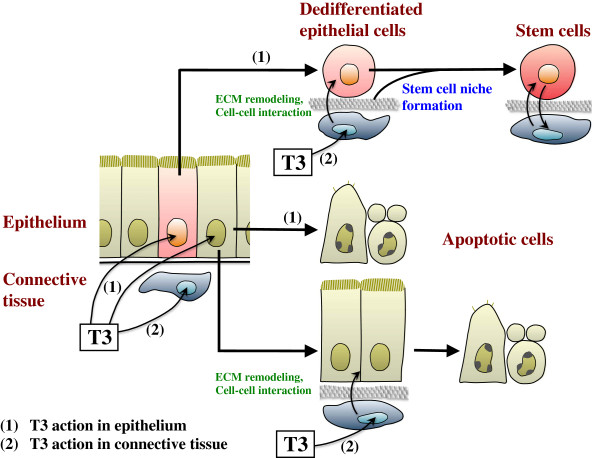
**A model for T3 actions for the metamorphic transformation of *Xenopus laevis *intestine.** During metamorphosis, T3 acts directly (**1**) on the larval epithelium as well as (**2**) on the rest of the intestine (the non-epithelium), mostly the connective tissue. Most of the larval epithelial cells are induced to undergo programmed cell death by either one of the two T3 actions: T3-induced cell autonomous apoptosis vs. apoptosis induced by ECM remodeling and cell-cell interaction due to T3 action in the non-epithelium. On the other hand, a small number of cells within the larval epithelium undergo dedifferentiation upon receiving the T3 signal. However, T3 action in these cells alone cannot induce the formation of adult stem cells, unless T3 action in the non-epithelium is also present. This suggests that T3-induced tissue interactions are required for the establishment of the stem cell niche, via ECM remodeling and cell-cell interaction, to enable the dedifferentiated epithelial cells to become stem cells. See [100] for details.

The above findings as well as other studies suggest that during metamorphosis, T3 signals in the larval epithelium and the rest of the intestine (the non-epithelium), mostly the connective tissue, have distinct effect on epithelial transformations (Figure 2). T3 action in either the epithelium or non-epithelium can cause larval epithelial apoptosis, the fate for most of the larval epithelial cells. Some of the larval epithelial cells, instead of undergoing apoptosis, begin to dedifferentiate and express Shh upon T3 induction. However, in the absence of T3 signaling in the non-epithelium, such cells cannot become stem cells or develop into the adult epithelium. T3 action in non-epithelium, most likely the connective tissue, is thus required for adult stem cell development, presumably through interactions with the epithelium and the establishment of the stem cell niche [100] (Figure 2).

#### A role of T3-induced MMPs in the connective tissue for epithelial transformation

As discussed above, the ECM remodeling is likely important for intestinal metamorphosis. EMC remodeling is largely mediated by matrix metalloproteinases (MMPs), a superfamily of Zn-dependent membrane-bound or secreted endopeptidases [101-108]. MMPs can affect cell fate and behavior through multiple mechanisms by cleaving protein components of the ECM as well as many non-ECM extracellular or membrane-bound proteins [101,104,109-111].

MMPs have long been implicated in amphibian metamorphosis. In fact, the first MMP, collagenase, was isolated as a collagen-degradation enzyme from the resorbing tadpole tail [112]. Gene expression studies have shown that essentially all MMPs analyzed so far are upregulated at least in some organs during amphibian metamorphosis [87,108,113-117]. Most of them are highly expressed in the intestine at the climax of metamorphosis [87,113-115], when ECM remodeling takes place [27]. The most studied among them is the MMP stromelysin-3 (ST3). ST3 is directly upregulated by T3 at the transcription level and is one of the first MMPs to be upregulated during natural intestinal remodeling, before the onset of epithelial cell death (by stage 58) [87,114-116,118]. Spatially, ST3 mRNA and protein are expressed in the fibroblasts within the connective tissue [114,119], suggesting that ST3 is one of the connective tissue genes that affect epithelial transformation by altering cell-cell and cell-ECM interactions. In support of this, when a functional blocking polyclonal antibody against *Xenopus laevis* ST3 was added to the T3-treated organ cultures of premetamorphic intestine, it inhibited T3-induced ECM remodeling and larval epithelial cell death [120]. In addition, after extended T3 treatment of the organ cultures, adult epithelial progenitor/stem cells were formed as clusters of cells or islets that expanded three dimensionally in T3-treated organ cultures. In the presence of the antibody, the T3-induced formation and proliferation of progenitor/stem cells still occurred. However, the adult epithelial islets expanded only laterally along the epithelium-connective tissue interface but failed to invade into the connective tissue [120], a process probably essential for adult epithelial fold formation during intestinal metamorphosis. Thus, ST3 is likely important, not only for ECM remodeling and larval cell death, but also for cell migration during the development of the adult intestine [120].

Complementary to the organ culture studies, transgenic overexpression of ST3 under the control of a heat shock-inducible promoter [121] in premetamorphic tadpoles resulted in larval epithelial cell apoptosis, activation of fibroblasts, and contacts between epithelial cells and fibroblasts in the intestine [122], mimicking changes during natural metamorphosis. In addition, the basal lamina separating the epithelium and connective tissue was also altered by transgenic expression of ST3 in premetamorphic tadpoles [122]. These and other findings indicate that ST3 expression alone is sufficient to induce some although not all T3-induced metamorphic program in the intestine [122], supporting an important role of T3 action in the connective tissue for epithelial transformations.

The exact mechanism by which ST3 functions remains to be investigated. Compared to other MMPs, ST3 has much weaker activities toward known ECM proteins but much higher activities toward a few non-ECM proteins such as α1-protease inhibitor, at least *in vitro*[123-125]. Interestingly, we have discovered that ST3 cleaves the 67 kd laminin receptor both *in vitro* and during metamorphosis [126-128] and more importantly, this cleavage correlates with T3-induced apoptosis in the intestinal epithelium and tail epidermis [127,128]. Transgenic overexpression of ST3 caused apoptosis in both tail epidermis and muscles [128]. However, little 67 kd laminin receptor could be detected in the tail muscles. Thus, ST3 may affect cell fate during metamorphosis through multiple mechanisms.

#### T3-induced signaling pathways mediating cell-cell interactions during adult epithelial development

A number of signal transduction pathways have been shown to be important for intestinal development in mammals. Among them are the Shh, WNT, BMP, and Notch pathways [3,4,129,130]. Some of them are involved in the development of the adult stem cells during intestinal metamorphosis. Shh is one of the first T3 response genes identified in the metamorphosing *Xenopus laevis* intestine [87,131]. It is directly induced by T3 at the transcription level as one of the earliest events during intestinal remodeling. Shh mRNA is highly upregulated at the climax of intestinal metamorphosis and then downregulated by the end of metamorphosis. It is specifically expressed in the intestinal epithelium and its expression correlates with the formation of the adult intestinal progenitor/stem cells [132]. We have recently analyzed the regulation of other components of this signaling pathway during natural and T3-induced intestinal remodeling [133]. These included Shh receptor proteins Patched (Ptc)-1 and Smoothened (Smo) and the three related, downstream transcription factors Gli1, Gli2 and Gli3. We found that like Shh, all were transiently up-regulated during intestinal metamorphosis. Interestingly, all were expressed in the connective tissues but not the epithelium. Thus, the epithelium-expressed Shh acts in a paracrine manner on the connective tissues during metamorphosis. In fact, by using intestinal organ cultures, we showed that overexpression of Shh upregulated the expression of Ptc-1, Smo, and Glis, even in the absence of T3, indicating that these components themselves are among Shh targets during intestinal remodeling [133]. More importantly, addition of recombinant Shh protein to the organ culture medium resulted in the activation of cell proliferation in both the epithelium and connective tissue in the absence of T3. In the presence of T3, developmental anomalies in the adult epithelium were caused by the addition of Shh. Thus, T3-upregulated expression of Shh regulates the development of the adult intestine, with high levels of Shh correlating with the formation and/or proliferation of the progenitor/stem cells and subsequent downregulation being important for the differentiation of the adult epithelial cells toward the end of metamorphosis [132].

In addition to Shh, bone morphogenetic protein-4 (BMP-4), a member of the TGFβ superfamily of signaling molecules, is also a T3 response gene [134]. BMP-4 is specifically expressed in the connective tissue and its expression temporally correlates with adult epithelial development in the *Xenopus laevis* intestine. Furthermore, BMPR-IA, a type I receptor of BMP-4, is expressed in both the developing connective tissue and progenitor/stem cells of the adult epithelium during metamorphosis, suggesting that BMP-4 affects both the connective tissue and adult epithelium during intestinal metamorphosis. Consistently, in intestinal organ culture, the addition of BMP-4 protein not only repressed cell proliferation in the connective tissue but also promoted differentiation of the adult epithelial cells [135]. Moreover, the addition of excessive Chordin, an antagonist of BMP-4, resulted in a decrease of adult epithelial cells in number and proliferation. This suggests that a certain level of BMP-4 may be necessary for the maintenance of the adult stem cells.

BMP-4 is a known target of Shh during mammalian gut development [136-138]. Like in mammals, Shh also induces the connective tissue-specific expression of BMP-4 during *Xenopus* metamorphosis [135]. Thus, the Shh and BMP-4 signaling pathways interact with each other to mediate epithelial–connective tissue interactions to affect intestinal metamorphosis. That is, Shh is directly induced by T3 in the adult progenitor/stem cells. The secreted Shh enhances the formation and/or proliferation of the progenitor/stem cells through yet unknown mechanism. At the same time it signals the connective tissue to increase the expression of Shh signaling components, thus providing a positive feedback on its own signaling, and to induce the expression of BMP-4. The connective tissue-derived BMP-4, in turn, represses cell proliferation in the connective tissue and promotes adult epithelial cell differentiation, both of which take place toward the end of intestinal metamorphosis [135].

## Conclusion

The mammalian adult intestinal stem cells have long been used as a model to study tissue renewal and stem cell maintenance. The establishment of the self-renewing system of the intestinal epithelium is largely conserved in vertebrates. It takes place during the postembryonic period when plasma T3 levels are high, and more importantly, depends on T3. In amphibians, this postembryonic period is the T3-dependent metamorphosis. Increasing evidence indicates that the formation of the self-renewing epithelium of the adult intestine involves the formation of the adult intestinal stem cells that are distinct from the larval/neonatal intestinal epithelial stem cells across vertebrate species. Analyses of *Xenopus* intestinal metamorphosis have led to the identification of many T3 response genes that are likely involved in the development of the adult stem cells and many such genes, such as those of the Shh and BMP-4 signaling pathways, likely have similar functions during the maturation of the adult intestine in mammals. A major future challenge will be to investigate the functions of such genes *in vivo*. The recent advancements in gene knockout studies in *Xenopus* by using gene-specific nucleases [139,140] undoubtedly enhance the value of the amphibian model for studying the molecular mechanisms of organ-specific adult stem cell development.

## Competing interests

The authors declare that they have no competing interest.

## Authors’ contributions

All authors participated in the writing of the review. All authors read and approved the final manuscript.
